# Defining the complex phenotype of severe systemic loxoscelism using a large electronic health record cohort

**DOI:** 10.1371/journal.pone.0174941

**Published:** 2017-04-19

**Authors:** Jamie R. Robinson, Vanessa E. Kennedy, Youssef Doss, Lisa Bastarache, Joshua Denny, Jeremy L. Warner

**Affiliations:** 1 Department of Biomedical Informatics, Vanderbilt University Medical Center, Nashville, TN, United States of America; 2 Department of General Surgery, Vanderbilt University Medical Center, Nashville, TN, United States of America; 3 Department of Internal Medicine, Stanford University, Stanford, CA, United States of America; 4 Yale University, New Haven, CT, United States of America; 5 Department of Medicine, Vanderbilt University Medical Center, Nashville, TN, United States of America; Universidad de Costa Rica, COSTA RICA

## Abstract

**Objective:**

Systemic loxoscelism is a rare illness resulting from the bite of the recluse spider and, in its most severe form, can lead to widespread hemolysis, coagulopathy, and death. We aim to describe the clinical features and outcomes of the largest known cohort of individuals with moderate to severe loxoscelism.

**Methods:**

We performed a retrospective, cross sectional study from January 1, 1995, to December 31, 2015, at a tertiary-care academic medical center, to determine individuals with clinical records consistent with moderate to severe loxoscelism. Age-, sex-, and race-matched controls were compared. Demographics, clinical characteristics, laboratory measures, and outcomes of individuals with loxoscelism are described. Case and control groups were compared with descriptive statistics and phenome-wide association study (PheWAS).

**Results:**

During the time period, 57 individuals were identified as having moderate to severe loxoscelism. Of these, only 33% had an antecedent spider bite documented. Median age of individuals diagnosed with moderate to severe loxoscelism was 14 years old (IQR 9.0–24.0 years). PheWAS confirmed associations of systemic loxoscelism with 29 other phenotypes, e.g., rash, hemolytic anemia, and sepsis. Hemoglobin level dropped an average of 3.1 g/dL over an average of 2.0 days (IQR 2.0–6.0). Lactate dehydrogenase and total bilirubin levels were on average over two times their upper limit of normal values. Eighteen individuals of 32 tested had a positive direct antiglobulin (Coombs’) test. Mortality was 3.5% (2/57 individuals).

**Conclusion:**

Systemic loxoscelism is a rare but devastating process with only a minority of patients recalling the toxic exposure; hemolysis reaches a peak at 2 days after admission, with some cases taking more than a week before recovery. In endemic areas, suspicion for systemic loxoscelism should be high in individuals, especially children and younger adults, presenting with a cutaneous ulcer and hemolysis or coagulopathy, even in the absence of a bite exposure history.

## Introduction

Systemic loxoscelism is a constitutional illness resulting from the bite of spiders of the genus *Loxosceles*, which is distributed worldwide. In parts of the United States, the *Loxosceles reclusa*, commonly referred to as the brown recluse spider, is endemic. Such bites commonly cause local necrosis, referred to as necrotic arachnoidism.[1] Systemic toxicity may also occur and in its mild form consists of nausea, vomiting, fever, chills, or arthralgia. In its more severe form, brown recluse bites may cause massive hemolysis, hemoglobinuria, acute renal failure, disseminated intravascular coagulation, and rarely death.[2–5] The most significant morbidity in systemic loxoscelism results from hemolysis and coagulopathy.[6, 7] Because hemolysis resulting from loxoscelism is uncommon, there is little known about its clinical manifestations, diagnosis, or outcomes.[8] Use of dapsone, once considered a treatment for systemic loxoscelism, has declined due to the suggestion of increased risk of hemolysis.[9] The underlying pathogenesis of systemic loxoscelism remains incompletely understood, but sphingomyelinase D, a component of the venom toxin, has been shown to have a central role in the process.[10–12] Recent literature suggests it causes both direct toxin-mediated hemolysis and complement-mediated erythrocyte destruction.[8, 11, 13] There is also an indication that hemolysis may be partly immune-mediated, given that a certain proportion of individuals reported in the literature have shown positive direct antiglobulin testing (DAT; Coombs’) for surface immunoglobulin G (IgG).[7]

The majority of brown recluse spider bite victims lack systemic symptoms, and severe systemic symptoms are even more rare.[14] In 2014, only 1,330 brown recluse spider bites were reported in the United States; of these, 481 individuals required treatment in a health care facility.[15] The brown recluse spider is endemic to the southeastern and Midwestern United States, and likelihood of envenomation outside of these areas is extremely low.[16, 17] Due to the limited geographic nature of the brown recluse and infrequent occurrence of systemic loxoscelism, there is little published on the clinical features and outcomes of these individuals.

In this study, we describe clinical characteristics and outcomes of the largest known cohort of individuals with systemic loxoscelism to date, leveraging our large de-identified electronic clinical data warehouse. We then performed a phenome-wide association study (PheWAS) of these individuals matched to a control population to identify key differences in ~1800 phenotypes between individuals who develop systemic loxoscelism and those who do not. PheWAS has previously been successfully applied to genomic and laboratory results with high validity to replicate known associations.[18–21] Our goal was to highlight clinical characteristics of this rare and potentially lethal illness, and to potentially uncover previously undocumented phenotypic associations.

## Materials and methods

A retrospective, cross-sectional study was performed to analyze suspected cases of loxoscelism at Vanderbilt University Medical Center (VUMC), a tertiary-care academic medical center, over a 20-year time span. VUMC consists of an adult and children’s hospital in the epicenter of the brown recluse geographical range.[17] Data collection was performed using the VUMC Synthetic Derivative (SD), a de-identified version of over 2.4 million patient electronic health records.[22] Dates are shifted at random +/- 365 days for each individual with relative time preservation. We identified all records with shifted dates between January 1^st^, 1995 and December 31^st^, 2015 containing any mention of “loxoscelism” in a clinical note, problem list, discharge summary, clinical communication, or letter. A two-person manual review of all flagged records was performed, and discrepancies were adjudicated by a third reviewer. This study was approved and designated as non-human subject research by the Institutional Review Board of Vanderbilt University Medical Center; therefore, consent was not necessary.

Individuals were manually excluded from the study if they lacked evidence of systemic loxoscelism, i.e., absence of fever, chills, abdominal pain, hemolysis or abnormal liver function tests. Individuals with moderate to severe loxoscelism were determined by the presence of a documented diagnosis of hemolysis or disseminated intravascular coagulation, need for blood transfusion, or hemodynamic instability.

Control individuals were extracted to be age-, sex-, and race-matched to the cases with a 50:1 control to case ratio.

Variable data extracted included demographics and clinical parameters, including length of hospital stay, intensive care unit (ICU) admission, need for hemodialysis, and mortality. Race was self-reported and extracted for population comparisons and matching of controls. We determined individuals who received dapsone prior to or during admission to VUMC. The presence of a toxicology consultation and/or operative intervention for the cutaneous lesion, if present, were also documented. Laboratory values obtained included hemoglobin (HGB), lactate dehydrogenase (LDH), total bilirubin, haptoglobin, creatinine, urinalysis, and DAT. We selected for laboratory data 1 week prior to and up 3 weeks after the first instance of either “loxoscelism” in a clinical document or ICD-9-CM billing code of 989.5 (toxic effect of venom). Descriptive statistics were performed, including mean or median with interquartile ranges for continuous variables or frequencies and percentages for categorical variables. Demographic differences between case and control groups were assessed using Chi-square or Fisher exact test, as appropriate. Wilcoxon rank sum test was used to compare ages of individuals with moderate-severe systemic loxoscelism to those with only cutaneous or mild systemic symptoms. PheWAS of cases versus controls was applied. PheWAS codes are aggregations of ICD-9-CM codes, as previously described.[19] All pairwise PheWAS comparisons were conducted using logistic regression with adjustment for age and sex. The minimum number of records to perform a test was 20 individuals in the combined case and control groups, with each individual having at least 2 instances of the PheWAS code. All statistical analyses were performed with R statistical software[23] using the PheWAS package and PheWAS code map version 1.2.[21]

## Results

The initial search found 373 possible cases with “loxoscelism” documented within the SD. After manual review and exclusion of subjects due to the lack of systemic loxoscelism, 57 individuals with moderate to severe loxoscelism were included in the final analysis. Of the excluded individuals, 90 were found to have cutaneous-only symptoms consistent with brown recluse spider bite, and 58 individuals had mild symptoms of systemic loxoscelism; the remainder had negation terms for loxoscelism (e.g., “this presentation is *not consistent* with loxoscelism.”). The control cohort consisted of 2,850 individuals.

### Demographic characteristics

Of the individuals identified, 54% were female and the majority Caucasian (37/57 individuals, 65%). A significantly larger portion of those with loxoscelism was African American (26%) compared to the SD population of ever-admitted individuals (15% African American [*p* = 0.02]).

**Fig 1:**
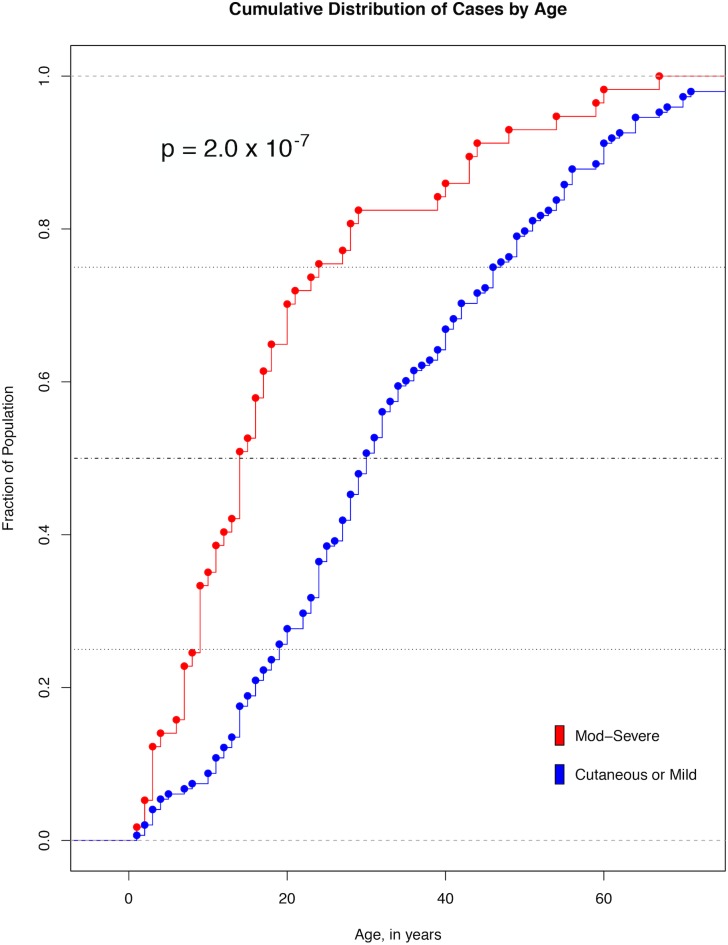
Cumulative Distribution of Cases by Age. Each point represents a case of either moderate-severe (mod-severe, red) loxoscelism or cutaneous/mild loxoscelism (cutaneous or mild, blue). The cumulative proportions of patients at or under a specific age are represented by each line. The median age of individuals with severe loxoscelism (14 years, IQR 9.0–24.0 yrs), was significantly lower than the median age (30 years old, IQR 19.0–46.0 yrs) of those identified with only cutaneous or mild systemic symptoms (*p* = 2.0 x 10^−7^).

The ages of those with moderate to severe loxoscelism were highly skewed towards children and young adults with 82.5% (47/57) of subjects under 30 years of age (Fig 1). The median age of included individuals was 14 years old (IQR 9.0–24.0 yrs), significantly younger (*p* = 2.0 x10^-7^) than the median age of those identified with only cutaneous or mild systemic symptoms (n = 148, median age 30 years old [IQR 19.0–46.0 yrs]). Admitted SD individuals were also on average older than those with loxoscelism (n = 667,990, median age 33 years old [IQR 12.0–58.0 yrs]).

All individuals presented with a cutaneous ulcer consistent with a brown recluse spider bite, but many did not recall an antecedent spider bite. According to the documentation present in the SD, only 33% (19 of 57) witnessed the spider and confirmed site of a brown recluse at the time of the envenomation. Ulcer location was most commonly on the upper extremity (27 of 57 individuals, 47%) or lower extremity (10 of 57 individuals, 18%). Further demographics and clinical characteristics are in Table 1.

**Table 1:** Clinical characteristics and outcomes of systemic loxoscelism.

	Loxoscelism Cohort (n = 57)	Reference
**Age**, median (IQR)	14.0 (9.0–24.0)	-
**Sex**, No. (%)		-
Male	26 (46%)	
Female	31 (54%)	
Unknown	0 (0%)	
**Race**, No. (%)		-
White	37 (65%)	
African American	15 (26%)	
Asian	1 (2%)	
Unknown/ Not Reported	4 (7%)	
Other	0 (0%)	
**Witnessed brown recluse spider bite**, No. (%)	19 (33%)	-
**Bite Location**, No. (%)		-
Upper extremity	27 (47%)	
Lower extremity	10 (18%)	
Chest	6 (11%)	
Back	6 (11%)	
Abdomen	4 (7%)	
Head/neck	3 (6%)	
Other	1 (2%)	
**Laboratory**, median (IQR)		
HGB (g/dL)	10.2 (8.4–11.7)	11.8–16.0
Lowest HGB (g/dL)	8.7 (5.7–10.5)	11.8–16.0
Change in HGB (n = 43, g/dL)	-3.1 (-1.8 to -5.6)	-
Average time to lowest HGB (n = 43, days)	2.0 (2.0–6.0)	-
LDH (unit/L)	529.0 (265.5–833.5)	<226
Highest LDH per individual (n = 37, unit/L)	739.0 (366.0–1344.0)	<226
Total Bilirubin (mg/dL)	2.9 (1.5–5.6)	0.2–1.2
Highest Total Bilirubin (n = 47, mg/dL)	4.3 (1.9–7.4)	0.2–1.2
Haptoglobin (mg/dL)	3	16–200
Lowest Haptoglobin (n = 23, mg/dL)	25.0 (2.5–129.5)	16–200
Creatinine (mg/dL)	0.8 (0.6–1.0)	0.70–1.50
Highest Creatinine (n = 54, mg/dL)	0.9 (0.6–1.2)	0.70–1.50
**Direct Antiglobulin positivity** (n = 32), No. (%)	18 (56%)	Negative
**Dapsone treatment**, No. (%)	2 (4%)	-
**Operative Intervention**, No. (%)	5 (9%)	-
**Toxicology Consult**, No. (%)	46 (80.7%)	-
**ICU Admission**, No. (%)	28 (49.1%)	-
**Length of Hospital Stay**, median days (IQR)	4.0 (2.0–5.0)	-
**Dialysis**, No. (%)	3 (5.3%)	-
**Mortality**, No. (%)	2 (3.5%)	-

IQR: Interquartile Range; HGB: Hemoglobin; LDH: Lactate Dehydrogenase; ICU: Intensive Care unit

### Clinical parameters

**Fig 2:**
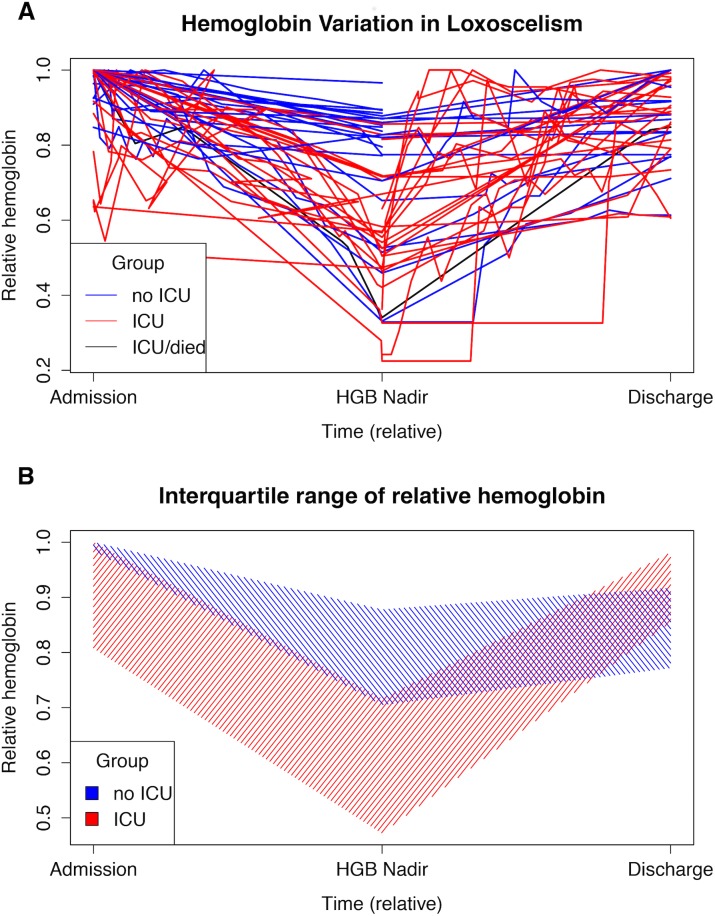
Hemoglobin Fluctuation During Loxoscelism. (A) Each line represents one individual with time and hemoglobin (HGB) graphed relative to the time point at the lowest HGB level (HGB Nadir) and the highest recorded HGB for each individual. Most HGB levels decline after admission and return to baseline. (B) ICU patients have lower interquartile ranges of HGB at presentation and at the HGB nadir, as compared to non-ICU patients. By the time of hospital discharge, the relative HGB level is similar between the two populations.

Laboratory parameters during the period with loxoscelism are in Table 1. Average HGB level at presentation was below normal (median 10.2 g/dL, reference 11.8–16.0). Furthermore, the average lowest HGB per case was significantly below normal at 8.7 g/dL (IQR 5.7–10.5). HGB decreased after admission in most individuals but gradually increased back to baseline, with or without supportive transfusion (Fig 2 Parts A-B). Relative decline in HGB was more severe for those admitted or transferred to the ICU during their hospitalization, compared to non-ICU areas. Not including the 12 individuals that arrived with their lowest recorded HGB, HGB level dropped an average of 3.1 g/dL from the first recorded level over an average of 2.0 days (IQR 2.0–6.0). There were 9 individuals with a decline in HGB of over 6 g/dL, of which 6 (67%) occurred 5–8 days after the first recorded value.

LDH (529.0 unit/L, reference < 226 unit/L) and total bilirubin (2.9 mg/dL, reference 0.2–1.2 mg/dL) were over two times their normal values. Median haptoglobin and creatinine levels were within normal range. Of the entire cohort, 32 individuals underwent DAT testing and 18 (56%) showed positivity. Of the 18 individuals with a positive DAT test, 9 (50%) were positive for C3 and IgG, 6 (33%) were positive for only IgG and 3 (17%) positive only for C3.

### PheWAS for loxoscelism phenotype

**Fig 3:**
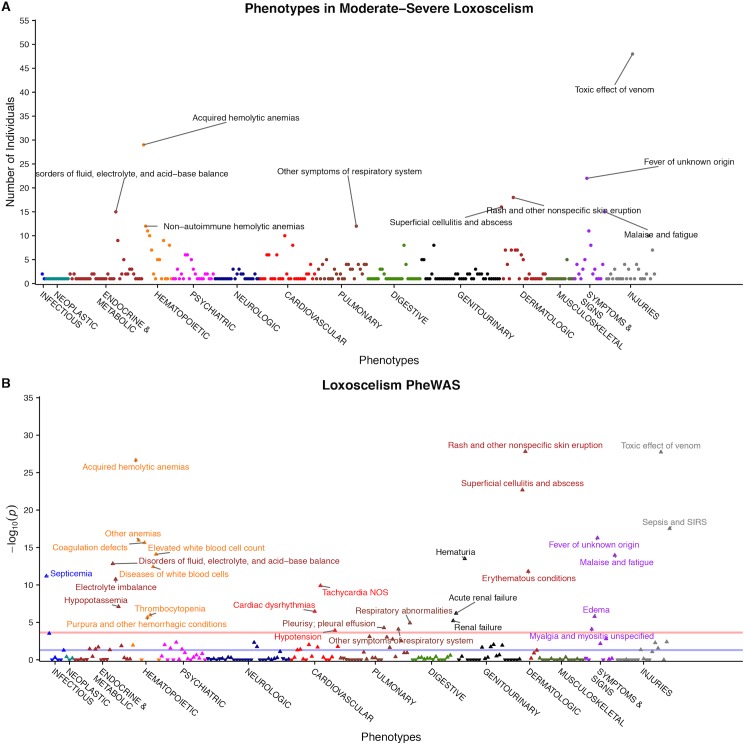
Phenotypes and PheWAS of Individuals with Moderate-Severe Loxoscelism. (A) Manhattan plot representing the number of individuals with moderate to severe loxoscelism with each phenotype. The most frequent phenotypes validated the loxoscelism definition and included the toxic effect of venom, acquired hemolytic anemia, fever of unknown origin, and rash/skin eruption. (B) PheWAS for moderate-severe loxoscelism. The blue line represents significance level without correction (*p* = 0.05). The red line is representative of the adjusted significance threshold using the Bonferroni correction for multiple comparisons (*p =* 1.2 x 10^−4^). 29 phenotypes showed a significant correlation (p < 1.2 x 10^−4^) with the loxoscelism phenotype when compared to controls.

A PheWAS for phenotypic associations with loxoscelism revealed many strong correlations including rash (*p* = 1.8 x 10^−28^), toxic effect of venom (*p* = 1.5 x 10^−28^), and hemolytic anemia (*p* = 2.0 x 10^−27^), which were also the most frequent phenotypes associated with individuals with loxoscelism (Fig 3 Parts A-B). These were clinical parameters used to assist in confirming the loxoscelism phenotype. Phenotypes that align with intravascular hemolysis, including coagulation defects (*p* = 2.3 x 10^−16^), hematuria (*p* = 3.1 x 10^−14^), and thrombocytopenia (1.1 x 10^−6^), were also strongly associated. The PheWAS analysis found strong associations between loxoscelism and superficial cellulitis/abscess (*p* = 2.1 x 10^−23^), sepsis (*p* = 3.1 x 10^−18^), and septicemia (*p* = 6.7 x 10^−12^), all possible correlations with infection. All statistically significant associations are in Table 2.

**Table 2:** Significant findings for PheWAS of loxoscelism (adjusted significance level, *p* < 1.2 x 10^−4^).

Clinical Phenotype	Cases in Loxoscelism only cohort (n = 57), No. (%)	Cases in Entire Population, No.	Controls in Entire Population, No.	OR (95% CI)	*p*-value
Rash and other nonspecific skin eruption	18 (32%)	57	2700	54 (27–110)	1.5x10^-28^
Toxic effect of venom	48 (84%)	51	2793	16745 (3714–130031)	1.8 x10^-28^
Acquired hemolytic anemias	29 (51%)	32	2674	2459 (700–12541)	2.0 x10^-27^
Superficial cellulitis and abscess	16 (28%)	65	2696	36 (18–75)	2.1 x10^-23^
Sepsis and SIRS	10 (18%)	22	2869	57 (23–144)	3.0 x10^-18^
Fever of unknown origin	22 (39%)	207	2484	17 (9–33)	5.3 x10^-17^
Other anemias	11 (19%)	93	2674	39 (16–94)	1.1 x10^-16^
Coagulation defects	10 (18%)	33	2790	33 (14–75)	2.3 x10^-16^
Elevated white blood count	8 (14%)	20	2736	64 (22–186)	8.1 x10^-15^
Malaise and fatigue	15 (26%)	113	2616	17 (8–35)	1.1 x10^-14^
Hematuria	8 (14%)	25	2630	36 (14–88)	3.1 x10^-14^
Disorders of fluid, electrolyte, and acid-base	15 (26%)	128	2632	12 (6–24)	1.5 x10^-12^
Diseases of white blood cells	9 (16%)	41	2736	23 (9–52)	3.7 x10^-13^
Erythematous conditions	7 (12%)	21	2765	44 (15–127)	1.6 x10^-12^
Septicemia	7 (12%)	26	2790	26 (10–65)	6.7 x10^-12^
Electrolyte imbalance	9 (16%)	57	2632	20 (8–47)	1.8 x10^-11^
Tachycardia	8 (14%)	44	2651	18 (7–40)	1.3 x10^-10^
Hypopotassemia	5 (9%)	28	2632	22 (7–65)	7.9 x10^-8^
Cardiac dysrhythmias	10 (18%)	118	2651	7 (3–15)	3.4 x10^-7^
Acute renal failure	5 (9%)	27	2812	14 (5–39)	6.1 x10^-7^
Thrombocytopenia	5 (9%)	35	2790	12 (4–32)	1.1 x10^-6^
Edema	5 (9%)	31	2825	13 (4–35)	1.7 x10^-6^
Purpura and other hemorrhagic conditions	5 (9%)	38	2790	11 (4–28)	2.2 x10^-6^
Renal failure	5 (9%)	35	2812	11 (3–29)	6.1 x10^-6^
Respiratory abnormalities	4 (7%)	23	2838	12 (3–34)	1.2 x10^-5^
Pleurisy, pleural effusion	4 (7%)	31	2735	11 (3–31)	5.3 x10^-5^
Myalgia and myositis unspecified	4 (7%)	32	2839	12 (3–37)	8.1 x10^-5^
Other symptoms of respiratory system	12 (21%)	229	2441	4 (2–8)	8.2 x10^-5^
Hypotension	4 (7%)	31	2834	9 (3–26)	1.2 x10^-4^

OR: Odds Ratio; SIRS: Systemic inflammatory response syndrome

### Treatment and outcomes

Clinical outcomes for individuals are reported in Table 1. All individuals were hospitalized except for one who died in the Emergency Department. The median length of hospital stay was 4.0 days (range, 1–28 days). Of the 57 subjects, 46 (80.7%) had a toxicology consult service assisting in diagnosis and management. Only 2 individuals (4%) received dapsone either prior to (per report) or during their hospital admission. Approximately half of individuals (49.1%) required initial admission or transfer to an ICU due to their critical condition and need for more intensive monitoring. Acute renal injury with a 2-fold increase in the creatinine level occurred in 6 individuals (10.5%). The increase in creatinine among these 6 individuals ranged from 0.7–6.9 mg/dL. Few individuals (3; 5.3%) required dialysis due to severe acute renal failure.

Only 2 individuals died during the 20-year study period from loxoscelism. One individual was a previously healthy 54-year-old man who developed severe hemolysis 5 days after a witnessed spider bite. He proceeded to multi-system organ failure with hemodynamic instability, renal and respiratory failure with ultimate cardiac arrest. The other individual was a previously healthy 3-year-old girl who developed signs of systemic loxoscelism within 6 hours of witnessed spider envenomation. She progressed to significant hematuria, anemia, thrombocytopenia, disseminated intravascular coagulopathy and shock within 19 hours of the bite resulting in death.[4]

## Discussion

This represents, to our knowledge, the largest cohort analyzed with moderate to severe loxoscelism to date. In contrast to prior published data consisting only of individuals with life-threatening hemolysis from severe loxoscelism[8], our cohort includes a wider phenotypic range consisting of all individuals with evidence of hemolysis. The large majority (70%) of individuals with systemic loxoscelism in our cohort were under 20 years of age. According to the 2014 annual report of the American Association of Poison Control Centers’ National Poison Data System, the majority (63%) of individuals who present in the United States with a brown recluse spider bite are 20 years or older. This suggests that although adults suffer from envenomation from brown recluse spiders more frequently (which was also seen in our data), children are subject to a much more severe reaction. Prior literature shows more frequent case reports of systemic loxoscelism occurring in pediatric individuals[4, 24–26], and this large retrospective review corroborates that children are at greater risk for systemic loxoscelism.

Hemolysis can occur in severe cases of loxoscelism[3], but may present differently than typical intravascular hemolysis. HGB is the most direct indicator of clinical severity in hemolytic disease[27] and its level can become extremely low (< 6 g/dL) in severe forms of loxoscelism, as seen in 15 (26%) of the individuals in our cohort. LDH is also known to be elevated during states of intravascular hemolysis[27] which was also demonstrated in this cohort with loxoscelism, whose average highest LDH was 3 times the upper limit of normal. Hyperbilirubinemia is also seen during hemolysis and can rise to > 4 mg/dL in severe acute hemolysis[27, 28], as was seen in our cohort of individuals. Haptoglobin is known to be decreased in periods of hemolysis[27]; however, our cohort only had 11 (19%) individuals with a haptoglobin less than normal and an average haptoglobin low within the normal range. Sterile tissue injury or infection can initiate a local inflammatory response that mobilizes a systemic acute phase reaction, resulting in the induction of genes encoding the acute phase plasma proteins, including haptoglobin.[29] Therefore, a state of inflammation from the brown recluse spider bite in many of the individuals may result in difficulty in interpretation of the haptoglobin level.[30]

In our review, it is important to note that the disease rarely progressed to renal failure. Only 6 individuals had a significant increase in creatinine level, 5 of which were identified with the PheWAS to have renal failure as well. Only 3 of these patients required dialysis. Furthermore, renal failure did not correspond with mortality in our series as the individuals who progressed to death did so in such a quick and extreme fashion that dialysis was not undertaken. Treatment of systemic loxoscelism is mainly supportive.[9] Steroids have been used to prevent kidney failure and hemolysis, but their efficacy is subject to debate.[9, 31] Other treatments that may be considered are dapsone, hyperbaric oxygen, and surgical excision.[9] Adverse side effects have been attributed to dapsone including hemolysis, anemia, and hyperbilirubinemia.[9] Only two individuals in our cohort received treatment with dapsone, both for short time periods. Although not statistically significant, one individual who received dapsone was an adult who developed severe hemolysis and subsequently died, whereas the other was a teenage male who suffered severe hemolysis that resolved. Omitting these individuals who received dapsone, the remainder of the hemolysis within our cohort cannot be attributed to treatment patterns of dapsone use.

Literature on rates of DAT positivity in systemic loxoscelism is scant; several small case series have reported high rates of positive DAT.[7, 32] Our large case series confirms DAT is positive in more than 50% of severely affected individuals, although this does not necessarily correlate with severity of disease. These prior reports demonstrated both IgG and C3 on the red blood cell surface, as is seen in a portion of our cohort with loxoscelism. Our results support the hypothesis that the anemia in loxoscelism can result from direct toxin-mediated erythrocyte damage, complement-mediated immune destruction, or both. Autoimmune hemolytic anemia[33] as well as other immune-mediated illnesses[34] are known to be associated with Human Leukocyte Antigen (HLA) type. HLA-DQ6 has shown in a small case series to have a negative association with a positive DAT result in individuals with hemolysis[33]; whether our cohort is enriched for this genotype is unknown.

As with most PheWAS analyses based on billing codes, all statistically significant phenotypes were of increased prevalence in the case population. We found that the PheWAS analysis recapitulated the described phenotype for systemic loxoscelism, and also suggested additional phenotypes of concern. Through PheWAS, some significant phenotypes were directly related to the defined definition of loxoscelism, whereas other phenotypes were likely due to secondary effects or potentially factors increasing risk for the disease process. Phenotypes of loxoscelism strongly replicated include toxic effect of venom (OR 16745, 95% CI 3714–130031) and hemolytic anemia (OR 2459, 95% CI 700–12541). The phenotype “toxic effect of venom” is mapped to a single ICD-9 code, 989.5, which applies to bites of venomous snakes, lizards, ticks, and spiders. We also found that the PheWAS analysis reconfirmed other known clinical findings in loxoscelism that were not within our loxoscelism case definition. These included rash or other nonspecific skin eruption (OR 54, 95% CI 27–110), hematuria (OR 36, 95% CI 14–88), coagulation defects (OR 33, 95% CI 14–75), malaise and fatigue (OR 17, 95% CI 8–35), and fever of unknown origin (OR 17, 95% CI 9–33).

Although hematuria was significant in PheWAS, hemoglobinuria was not. Hematuria and hemoglobinuria are both known findings in systemic loxoscelism, with hematuria occurring almost invariably in severe disease.[35–39] Urine dipstick analysis for blood is typically the initial screening test for hematuria or hemoglobinuria. In subsequent testing, the presence of red blood cells on microscopic urinalysis are indicative of hematuria. However, if the blood is detectable on a dipstick with no or very few microscopically visible RBCs, hemoglobinuria or myoglobinuria is suggested.[40] It is possible that misclassification of these billing diagnosis codes occurred, resulting in a stronger phenotypic association with hematuria. We did note in our cohort that hematuria, sometimes gross hematuria, was a present in patients with very severe forms of loxoscelism.

Several phenotypes more likely to be secondary effects of loxoscelism were also found to be significant in the PheWAS analysis. In particular, there appears to be a strong signal for septicemia (OR 26, 95% CI 10–63), sepsis and systemic inflammatory response syndrome (OR 56, 95% CI 22–140)–suggesting that bacterial superinfection of the local wound site and/or iatrogenic infections are an important consideration in this population. There is also a strong signal for cardiac dysrhythmias (OR 7, 95% CI 3–14) and electrolyte imbalance (OR 20, 95% CI 8–46), suggesting that the systemic loxoscelism process or secondary effects (massive fluid resuscitation, renal failure, etc.) lead to significant issues requiring intensive medical management. With the well-defined phenotype captured by PheWAS analysis, it is possible to construct phenotype risk scores that could capture individuals that may not have been formally diagnosed; this remains the subject of future work.[41]

There are several limitations to our study. This study was undertaken at a single institution and may not be generalizable to others, especially institutions within other countries in North and South America where antivenom is an option for medical treatment. Although the use and efficacy of antivenom is controversial, with studies indicating potentially limited capacity of the medication to neutralize the systemic effects of loxoscelism due to delayed presentation of illness, further research in this area is needed.[42] Our case identification relied on the use of the word “loxoscelism” in clinical notes; this word is uncommon in the medical jargon and could be hypothetically misspelled. However, a preliminary investigation using the keywords “brown recluse” resulted in a large false positive rate. Another limitation is that many of the cases were referred to VUMC for tertiary-level care and had very little preceding or subsequent history in the SD, limiting our ability to evaluate for long term sequelae in sufferers of systemic loxoscelism. Most importantly, our retrospective chart review was limited by the fact that systemic loxoscelism is a clinical diagnosis that is made upon the presence of systemic symptoms, a cutaneous lesion consistent with a brown recluse spider bite, and clinical presentation and history. There is no confirmatory test for its diagnosis. We believe our criteria to require laboratory markers of hemolysis and documented diagnosis of loxoscelism was the most accurate approach to determining the true prevalence of loxoscelism within our institution. Furthermore, review was performed by physicians to ensure accuracy. More stringent criteria, such as requirement that the spider responsible for envenomation is captured and confirmed to be a brown recluse, would likely lead to a profound underestimation of the actual occurrence of systemic loxoscelism in endemic areas. Lastly, as the severity of loxoscelism occurs along a spectrum without a clear definition of what determines severe disease, we did not distinguish between moderate and severe loxoscelism, including in our cohort all individuals with systemic signs of hemolysis or disseminated intravascular coagulation, need for blood transfusion, or hemodynamic instability. This allowed for greater inclusion of individuals affected by the disease; however, it also led to increased variability in our phenotype.

In conclusion, systemic loxoscelism is a rare occurrence, but within a region endemic to brown recluse spiders, multiple individuals present yearly with moderate to severe loxoscelism. A portion of these individuals develops moderate to severe hemolysis. Although children and possibly African Americans appear to be at increased risk, it remains unclear what specific risk factors correlate with disease severity.
